# LC‐ESI‐QTOF/MS characterization of antimicrobial compounds with their action mode extracted from vine tea (*Ampelopsis grossedentata*) leaves

**DOI:** 10.1002/fsn3.2679

**Published:** 2022-01-19

**Authors:** Muhammad Umair, Tayyaba Sultana, Zhu Xiaoyu, Ahmed M. Senan, Saqib Jabbar, Labiba Khan, Muhammad Abid, Mian Anjum Murtaza, Dhama Kuldeep, Niyazi A. S. Al‐Areqi, Lu Zhaoxin

**Affiliations:** ^1^ College of Food Science and Technology Nanjing Agriculture University Nanjing China; ^2^ College of Public Administration Nanjing Agriculture University Nanjing China; ^3^ Food Science Research Institute (FSRI) National Agricultural Research Centre Islamabad Pakistan; ^4^ Institute of Food and Nutritional Sciences Pir Mehr Ali Shah, Arid Agriculture University Rawalpindi Rawalpindi Pakistan; ^5^ Institute of Food Science and Nutrition University of Sargodha Sargodha Pakistan; ^6^ Division of Pathology ICAR‐Indian Veterinary, Research Institute Izatnagar India; ^7^ Department of Chemistry Faculty of Applied Science Taiz University Taiz Republic of Yemen

**Keywords:** 5,7,8,3,4‐pentahydroxyisoflavone, action mode, antimicrobial activity, bactericidal kinetics, LC‐ESI‐QTOF/MS, vine tea

## Abstract

Vine tea (*Ampelopsis grossedentata*) is a tea plant cultivated south of the Chinese Yangtze River. It has anti‐inflammatory properties and is used to normalize blood circulation and detoxification. The leaves of vine tea are the most abundant source of flavonoids, such as dihydromyricetin and myricetin. However, as the main bioactive flavonoid in vine tea, dihydromyricetin was the main focus of previous research. This study aimed to explore the antibacterial activities of vine tea against selected foodborne pathogens. The antimicrobial activity of vine tea extract was evaluated by the agar well diffusion method. Cell membrane integrity and bactericidal kinetics, along with physical damage to the cell membrane, were also observed. The extract was analyzed using a high‐performance liquid chromatography‐diode array detector (HPLC‐DAD), and the results were confirmed using a modified version of a previously published method that combined liquid chromatography and electrospray‐ionized quadrupole time‐of‐flight mass spectrometry (LC‐ESI‐QTOF/MS). Cell membrane integrity and bactericidal kinetics were determined by releasing intracellular material in suspension and monitoring it at 260 nm using an ultraviolet (UV) spectrophotometer. A scanning electron microscope (SEM) was used to detect morphological alterations and physical damage to the cell membrane. Six compounds were isolated successfully: (1) myricetin (C_15_H_10_O_8_), (2) myricetin 3‐O‐rhamnoside (C_21_H_20_O_12_), (3) 5,7,8,3,4‐pentahydroxyisoflavone (C_15_H_10_O_7_), (4) dihydroquercetin (C_15_H_12_O_7_), (5) 6,8‐dihydroxykaempferol (C_15_H_10_O_8_), and (6) ellagic acid glucoside (C_20_H_16_O_13_). Among these bioactive compounds, C_15_H_10_O_7_ was found to have vigorous antimicrobial activity against *Bacillus* *cereus* (AS11846) and *Staphylococcus aureus* (CMCCB26003). A dose‐dependent bactericidal kinetics with a higher degree of absorbance at optical density 260 (OD_260_) was observed when the bacterial suspension was incubated with C_15_H_10_O_7_ for 8 h. Furthermore, a scanning electron microscope study revealed physical damage to the cell membrane. In addition, the action mode of C_15_H_10_O_7_ was on the cell wall of the target microorganism. Together, these results suggest that C_15_H_10_O_7_ has vigorous antimicrobial activity and can be used as a potent antimicrobial agent in the food processing industry.

## Graphical abstract

This study explored the antimicrobial utility of vine tea (*Ampelopsis grossedentata*) against disease‐causing foodborne pathogens.

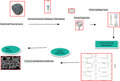

## 1 INTRODUCTION

Globally, six million people suffer from foodborne illness caused by pathogenic microorganisms (Basak et al., 2021). Food spoilage occurs due to the microbial growth and enzymes action from microbia (Delshadi et al., 2021). Food quality and safety are the chief concerns in the food industry (Cui, Li, et al., 2021). If these concerns are not addressed, food poisoning and other health issues arise (Yao et al., 2021).

Chemical preservatives have been used to prevent and control microbial growth in food (Hussain et al., 2021). However, there is an increasing consumer concern about the risks associated with the use of synthetic preservatives (El‐Saber Batiha et al., 2021). Many studies have explored natural plant sources as antimicrobial agents (El‐Saber Batiha et al., 2021; Molet‐Rodríguez et al., 2021; Varghese et al., 2020). The World Health Organization's traditional medicine centers indicated that 80% of the total bioactive compounds used for ethno‐medical purposes were derived from plant sources (Sivagurunathan Moni et al., 2021; Sopalun et al., 2021).

Recent works have focused on various functional aspects of dihydromyricetin, such as antimicrobial properties (Cui, Li, et al., 2021; Wu et al., 2017; Xiao et al., 2019), antioxidant capacity (Xie et al., 2019), flavor infusion (Carneiro et al., 2020), stability and stereo‐specific action (Umair et al., 2020), pharmacological activities (Muhammad et al., 2018), the bioavailability of bioactive compounds (Sun et al., 2021), interaction with iron (Wang et al., 2020), and its optimized extraction (Muhammad et al., 2017) by using response surface methodology. The proposed antimicrobial mechanism of dihydromyricetin in previous reports (Farhadi et al., 2019; Liang et al., 2020; Shevelev et al., 2020; Zhang et al., 2018) is comprises on; the membrane permeability, functional changes in the cell membrane, cytoplasmic membrane damage, blockage in the energy metabolism, retardation in the nucleic acid synthesis process, porin inhibition on the cell membrane, and weakening of the pathogenicity (Farhadi et al., 2019; Xie et al., 2019; Xie et al., 2015).

Secondary metabolites are increasingly preferred as natural preservatives in food due to their antibacterial activity against a variety of microorganisms. Thus, it is critical to find safe and natural antibacterial chemicals that will inhibit foodborne microorganisms and improve food quality. Yet, so far, only a few studies have been done to explore the antimicrobial potential of vine tea extract, so this study aims to explore the antimicrobial activity of the methanolic extract prepared from vine tea leaves. To evaluate the isolate separation, a high‐performance liquid chromatography‐diode array detector was employed (HPLC‐DAD). To validate the identity of these isolates, we used a well‐established liquid chromatography method in conjunction with triple‐quadrupole time‐of‐flight mass spectrometry with electrospray ionization in positive and negative modes. This study also focused on the antimicrobial activities of various isolates using the agar well diffusion method and its action mode against selected microorganisms.

## 2 MATERIAL AND METHODS

### 2.1 Sample preparation

Vine tea (*Ampelopsis grossedentata*) leaves were acquired from Moyeam Group Co., Ltd., in Changsha, Hunan, China (Grade 4 according to botanical classification). The voucher specimen of *A*. *grossedentata* was verified and deposited at Nanjing Agricultural University's College of Food Science and Engineering in Nanjing, Jiangsu, China. The material was air‐dried in the dark at room temperature. Dried vine tea leaves were stored in a tightly sealed jar until further use.

### 2.2 Isolation of compounds from vine tea extract

Dried ripe leaves of the vine tea plant were ground to fine powder. Fifty grams (50 g) of dried leaf powder was extracted with 500 ml of 75% aqueous methanol in 4 h at 25°C. The extract was then filtered through a filter paper to remove leaves and collect the extractant. The extractant was further concentrated under reduced pressure using a rotary evaporator (CCA‐20 Low Temp Cooling Liquid Circulating Pump; RE‐5299 Yarong) and stored for further phytochemical analysis. Sephadex LH‐20 (Pharmacia biotech) resin was used for the preparative purification and removal of steroids, terpenoids, and lipids from methanol extract. The methanol extract was then chromatographed by eluting with the solvent mixture (methanol and chloroform) with increasing methanol up to 90%. All fractions were collected separately and used for further analysis.

### 2.3 HPLC and LC‐ESI‐QTOF spectroscopic mass analysis

The vine tea extract was separated into different components using HPLC‐DAD, and the isolated components were then validated using a modified version of the current method of liquid chromatography coupled with electrospray ionization quadrupole time‐of‐flight mass spectrometry (LC‐ESI‐QTOF/MS). Prior to HPLC‐DAD analysis, the material was filtered using a filter membrane (0.45 µm, 13 mm). A 10 µl sample was injected into the separating column C18 X‐Bridge™ (OBDTM, 150 mm × 19 mm, 5 m) with a mobile phase mixture of solvent A, acidified methanol (0.1% trifluoroacetic acid (TFA)), and solvent D (0.1% TFA in water). The gradient program was conducted for 55 min under the following conditions: 0–10 min, 0%–40% A; 10–15 min, 40%–50% A; 15–25 min, 50%–75% A; 25–30 min, 75%–90% A; 30–40 min, 90%–100% A; 40–45 min, 100%–30% A; 45–50 min, 30%–2% A; and 50–55 min, 2% isocratic. The mobile phase flow rate was set to 0.8 ml/min, and the elution was measured at 220, 259, 289, and 340 nm at a rate of 1.25 scans/s (peak width = 0.2 min).

A TSQ‐Quantum Access MAX LC/MS system (Thermo) was used for the LC‐ESI‐QTOF/MS analysis. Agilent 1200 HPLC and 6520 QTF‐MS systems (Agilent Technologies) equipped with heated electrospray ionization (ESI) sources were used for the high‐resolution mass spectrometry study. Positive and negative modes of identification were used, with mass spectra (*m*/*z*) ranging from 100 to 1200. The mass spectrometry conditions were as follows: The column and conditions were identical to those described in HPLC‐DAD with the exception of the injection sample volume (20 µl); the spray voltage source was set to 4.0 kV, the heated transfer capillary temperature was set to 350°C, and the argon vaporizer temperature was set to 200°C. With a collision pressure of 1.0, the sheath gas was set to 40 arbitrary units and the auxiliary gas to 5 arbitrary units.

### 2.4 Antimicrobial activity assay

Different microorganisms were chosen for antimicrobial studies such as *Staphylococcus aureus* (CMCCB26003), *Bacillus cereus* (AS11846), *Bacillus pumilus* (CMCC63202), two gram‐negative bacteria, *Escherichia coli* (ATCC25922) and *Pseudomonas fluorescens* (AS11802), and three fungal strains, *Fusarium moniliforme* (30174), *Fusarium graminearum* (2021), and *Aspergillus flavus* (CICC2062). These microorganisms were collected by the China Committee for Culture Collection of Microorganisms (CMCC).

The antimicrobial activity was determined by the agar well diffusion method (Valgas et al., 2007). Different concentrated solutions (256, 128, 64, 32, and 16 µg/ml) of each isolated compound (1–6) were tested. All bacterial strains were grown in LB (20% agar) medium for at least 24 h at 37°C, and molds were grown in potato dextrose agar (PDA) medium for 72 h at 28°C (200 g/L; agar 20 g/L; dextrose 10 g/L at 7.0 pH). Nisin (32 g/ml) and tetracycline (32 g/ml) were used as positive controls, whereas methanol solution was used as a negative control. Triplicates of the assay were performed. Additionally, the minimum inhibitory concentration (MIC) and minimum bactericidal concentration (MBC) were determined using the technique described in Zhao et al. (2013). A solution of Tween‐80 (5% w/v) was added to the antibacterial agent. Tween‐80 was generated in this experiment at concentrations ranging from 16 to 256 mg/ml using a twofold serial dilution of sterile nutritional broth medium. Six compounds were administered to identical bacterial strains in 96‐well microplates at the same dose and volume. In addition, the plates were incubated at 37°C for 24 h, during which time visible turbidity was observed inside the plates. MBC was measured by subculturing 10 ml of the MIC solution on PDA for 24 h at 37°C.

### 2.5 Determination of extracellular activity

#### 2.5.1 Cell membrane integrity

The integrity of *B*. *cereus* (AS11846) bacterial cells was evaluated by determining component leakages in supernatants using optical density at 260 nm (OD_260_), as described by Cui et al. (2015). The supernatant from each sample was collected and tested for absorbance at OD_260_ at different time points. Thus, exponentially growing bacteria were collected and centrifuged at 5000 *g* for 5 min at 4°C before being added to phosphate buffer (PBS 0.1 M, pH 7.0). Finally, the microbial cells were treated for 1, 2, 3, and 4 h with 5,7,8,3,4‐pentahydroxyisoflavone (C_15_H_10_O_7_; 32 g/ml), whereas the bacterial suspension without the extracted chemical (compound 3 in this case) served as a control.

#### 2.5.2 Kinetics of bactericidal activity

The kinetics of bactericidal activity of the compound C_15_H_10_O_7_ against *B*. *cereus* (AS11846) was determined as follows: The exponential phase bacterial strain of *B*. *cereus* (AS11846) was taken for experiments. The antimicrobial activity was measured by colony‐forming unit (CFU) at 600 nm (OD_600_), while the bacterial suspensions (1 × 10^5^ CFU/ml) were grown overnight in NB at a temperature of 37°C and a rotational speed of 180 rpm for 24 h. Tetracycline was administered as a positive control throughout the treatment. After 1, 4, 8, 16, and 24 h, aliquots were collected and diluted with a twofold serial dilution with 0.85% (w/v) saline solution. Aliquots were then cultured in triplicate on Mueller‐Hinton agar plates. After 24 h of incubation at 37°C, several CFUs were counted. More than three log reductions in CFU/ml compared with the initial concentration are regarded as a bactericidal effect (Duffy et al., 2018).

#### 2.5.3 Determination of physical damage in the cell membrane through SEM

The SEM examination was carried out using the method of Han et al. (2017). Compound 3 was treated with *B*. *cereus* (AS11846) and *E*. *coli* (ATCC25922) cell suspensions at a concentration of 32 g/ml (1MIC) for 1, 2, 4, and 8 h, followed by centrifugation at 5000 *g* for 5 min at 4°C (Eppendorf centrifuge 5804R AG). These were then suspended in phosphate‐buffered saline (PBS; pH 7.0), the pallets were washed three times with buffer, and the separated compounds were reacted to a final concentration of 32 g/ml prior to being transferred to the cover slip. The control sample did not include the C_15_H_10_O_7_ molecule. Cells mounted on slides were fixed with a glutaraldehyde solution (2.5% w/v). The SEM (SU‐8010, Hitachi) showed physical damage to the cell membrane at 30 kV of the electric beam.

### 2.6 Statistical analysis

All analyses were performed in triplicate. The data were presented as mean ± standard deviation (SD). Microsoft Excel software (Microsoft, USA) was used to generate graphics. One‐way analysis of variance and *t‐*tests were used at the level of significance of *p* < .05. The Statistical Package for the Social Sciences (SPSS) 17.0 was used to examine all of the data.

## 3 RESULTS AND DISCUSSION

### 3.1 HPLC and LC‐ESI‐QTOF spectroscopic mass analysis

**FIGURE 1:**
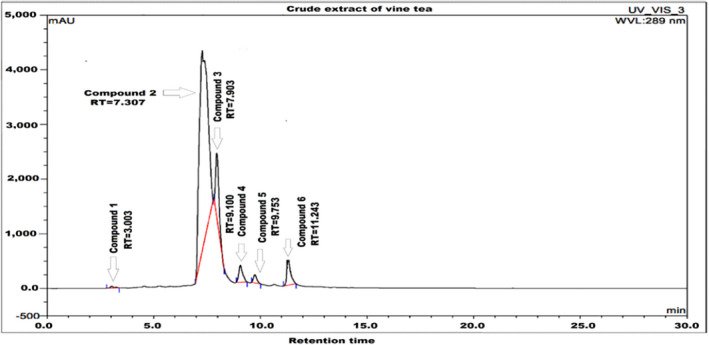
High‐performance liquid chromatography spectra of compounds 1–6 from vine tea extract

**FIGURE 2:**
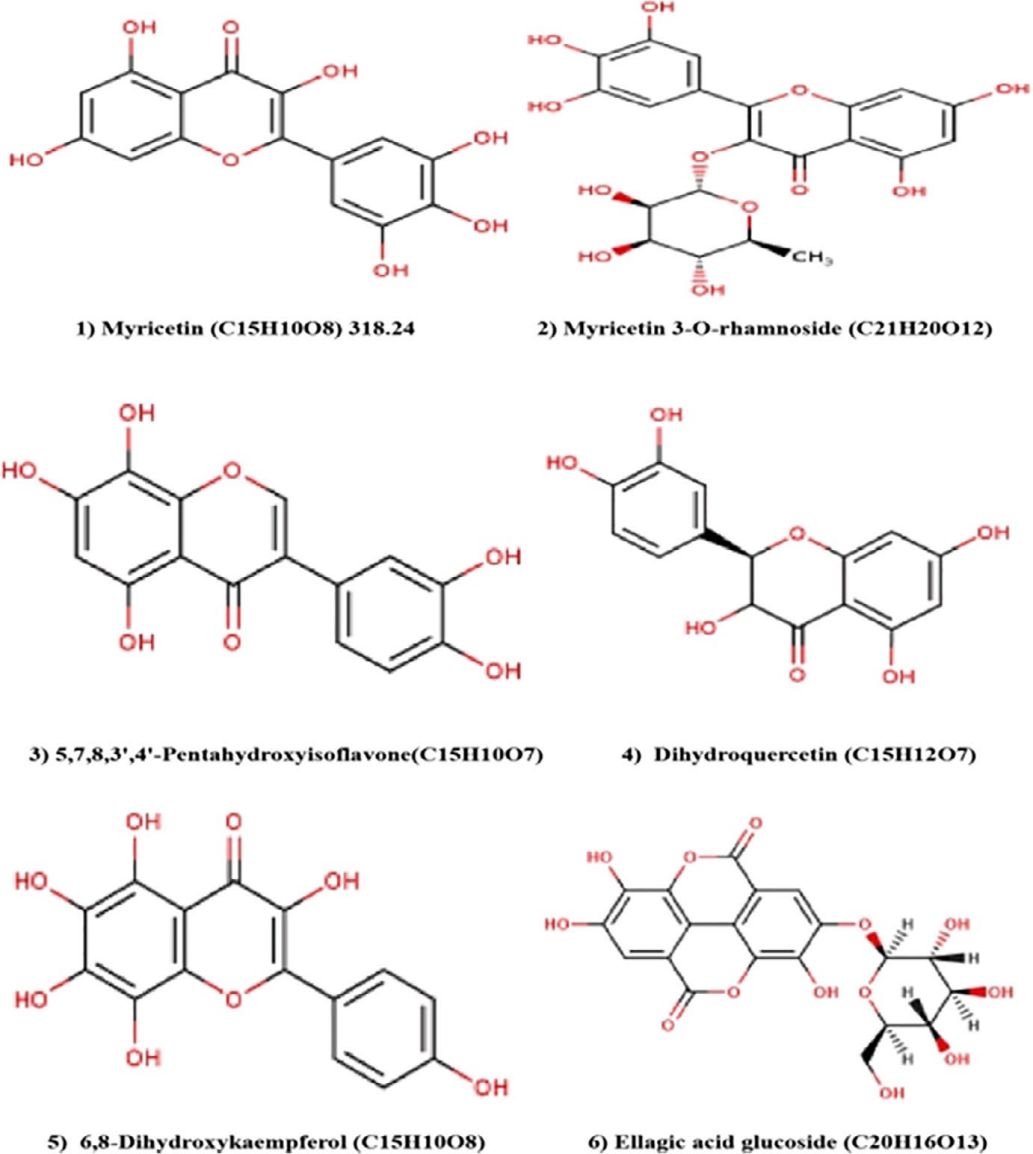
Chemical structures of compounds 1–6 isolated from vine tea extract

The results of the HPLC‐DAD analysis were used for the analytical characterization of the methanolic vine tea extract phytoconstituents. Separation was based on various parameters like retention time, physical properties, molecular weight, and molecular formula. Six bioactive products were found in vine tea extract by HPLC‐DAD (Figure 1). Different spectral investigations were employed to interpret the isolated molecules and to authenticate all isolated compounds. The six phytoconstituents were identified as follows: (1) myricetin (C_15_H_10_O_8_), (2) myricetin 3‐O‐rhamnoside (C_21_H_20_O_12_), (3) 5,7,8,3,4‐pentahydroxyisoflavone (C_15_H_10_O_7_), (4) dihydroquercetin (C_15_H_12_O_7_), (5) 6,8‐dihydroxykaempferol (C_15_H_10_O_8_), and (6) ellagic acid glucoside (C_20_H_16_O_13_) as mentioned in Table 1. Previous reports have published and reported some of these compounds from vine tea but not all six in the same plant, such as C_15_H_10_O_8_ (Fan et al., 2012; Zhang et al., 2020; Zhao et al., 2021; Long et al., 2019), C_21_H_20_O_12_ (Ma et al., 2020; Zhang et al., 2020), C_15_H_10_O_7_ (Cui et al., 2021; Hosny & Rosazza, 1999), C_15_H_12_O_7_ (Gao et al., 2017), C_15_H_10_O_8_ (Kim et al., 2019; Zhou et al., 2013), and C_20_H_16_O_13_ (Kuba‐Miyara et al., 2012; Yu et al., 2021). The chemical structure of all six isolated compounds is enlisted in Figure 2.

**TABLE 1:** Compounds 1–6 isolated and purified by using LC‐ESI‐QTOF/MS

No	Chemical name	formula	Retention time (Min)	Mass spectra calc. (*m*/*z*)	Mass spectra obs. (*m*/*z*)	ESI‐MS	Error	UV (*I*_max_)
1	Myricetin	C_15_H_10_O_8_	3.003	318.235	317.030	[M‐H]^‐^	0.198	220
2	Myricetin3‐0‐rhamnoside	C_21_H_20_O_12_	7.307	464.376	463.088	[M‐H]^‐^	0.282	254
3	5,7,8,3',4'‐pentahydroxyisoflavone	C_15_H_10_O_7_	7.903	302.235	301.035	[M‐H]^‐^	0.193	220
4	Dihydroquercetin	C_15_H_12_O_7_	9.100	304.058	303.051	[M‐H]^‐^	0.001	289
5	6,8‐Dihydroxykaempferol	C_15_H_10_O_8_	9.753	318.037	317.030	[M‐H]^‐^	0.001	254
6	Ellagic acid glucoside	C_20_H_16_O_13_	11.243	464.059	465.048	[M+H]^+^	0.016	254

**FIGURE 3:**
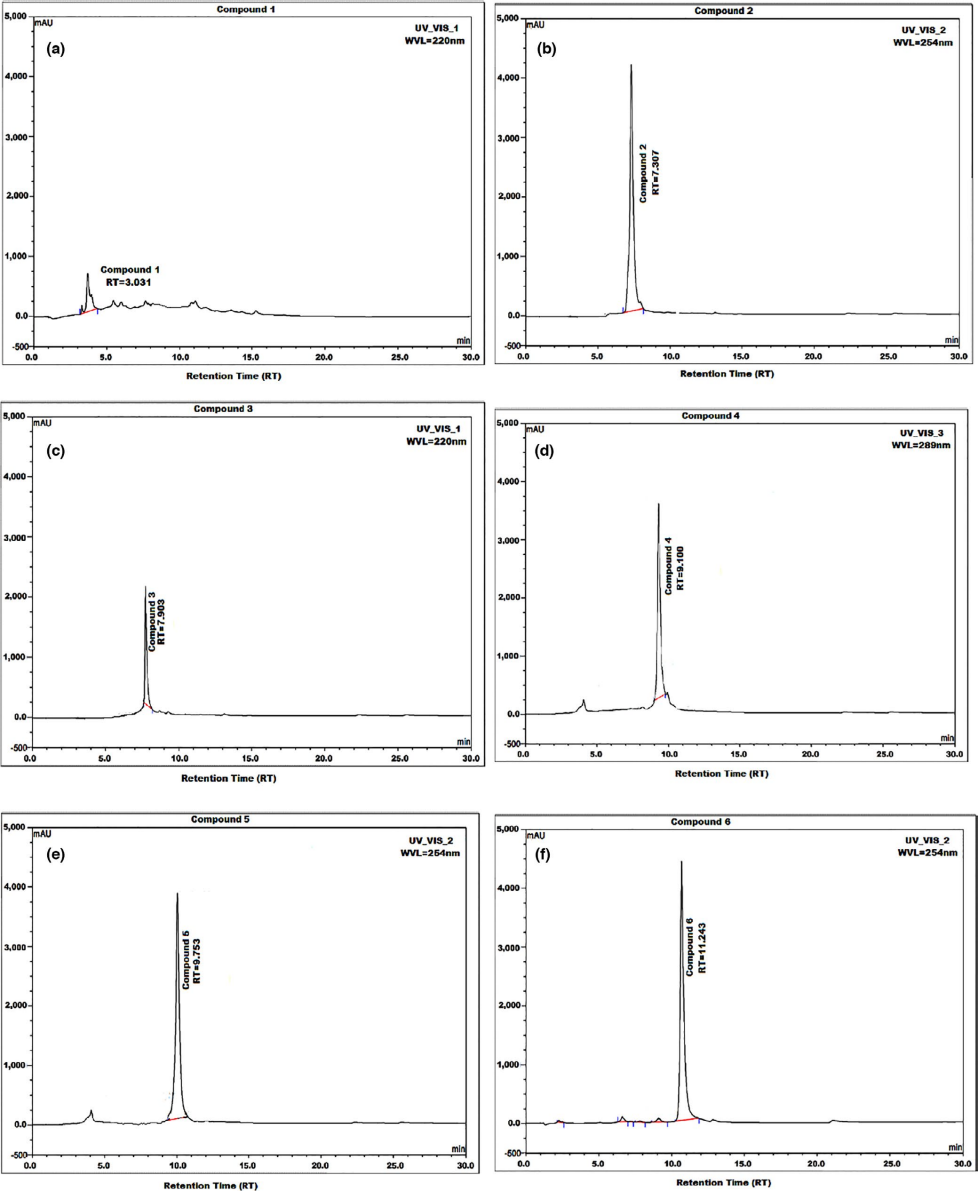
(a–f) High‐performance liquid chromatography spectra of compounds 1–6 from vine tea extract

Next, a MIC was established for six isolated products against all tested microorganisms as presented in Table 2. Based on the highest antimicrobial activity of compound 3 (C_15_H_10_O_7_) against all tested microorganisms, which is reported for the first time in this plant, it is further selected for a detailed study. The molecular formula of compound 3 was identified as C_15_H_10_O_7_ with a molecular weight of 301.03597 and was obtained as brown‐yellow solid particles that are soluble in water and methanol (Figure 3).

**TABLE 2:** Zone of inhibition activity of isolated compounds (1–6) against gram‐positive bacteria, gram‐negative bacteria, and fungi

Sample	*S. aureus* ATCC25923	*B. pumilus* CMCC63202	*B. cereus* AS11846	*E. coli* ATCC25922	*P. fluorescens* AS11802	*F. graminearum* CICC2021	*F. moniliforme* CICC30174	*A. flavus* CICC2062
	mm	mm	mm	mm	mm	mm	mm	mm
1	19.30 ± 0.81^b^	17.41 ± 0.16^b^	15.42 ± 0.64^c^	16.12 ± 0.25^c^	17.54 ± 0.12^b^	15.43 ± 0.12^b^	14.34 ± 0.39^b^	13.28 ± 0.13^b^
2	15.20 ± 0.44^e^	16.71 ± 0.10^c^	16.42 ± 0.44^b^	17.52 ± 0.15^b^	17.12 ± 0.23^b^	12.62 ± 0.15^c^	11.58 ± 0.33^d^	12.19 ± 0.32^c^
3	22.20 ± 0.17^a^	21.37 ± 0.32^a^	25.42 ± 0.12^a^	24.21 ± 0.10^a^	22.12 ± 0.25^a^	19.78 ± 0.22^a^	18.54 ± 0.12^a^	19.78 ± 0.22^a^
4	17.55 ± 0.78^c^	16.57 ± 0.40^c^	13.56 ± 0.32^e^	17.32 ± 0.19^b^	16.54 ± 0.12^c^	NA	NA	NA
5	16.27 ± 0.28^d^	15.97 ± 0.30^d^	16.03 ± 0.11^b^	16.78 ± 0.22^c^	16.12 ± 0.25^c^	11.42 ± 0.38^d^	10.54 ± 0.17^e^	13.78 ± 0.33^b^
6	19.20 ± 0.19^b^	17.57 ± 0.10^b^	15.22 ± 0.21^c^	14.12 ± 0.25^d^	16.78 ± 0.22^c^	12.42 ± 0.12^c^	12.54 ± 0.14^c^	13.38 ± 0.20^b^
7	13.1 ± 0.22^f^	14.42 ± 0.28^e^	16.20 ± 0.23^b^	NA	NA	NA	NA	NA
8	15.51 ± 0.26^e^	13.47 ± 0.41^f^	14.22 ± 0.67^d^	NA	NA	NA	NA	NA

Note

Values with different letters (a–f) in the same column are significantly different from each other; number in columns 1–6 represents the six isolated compounds: 1 = Myricetin, 2 = Myricetin 3‐0‐rhamnoside, 3 = 5,7,8,3',4'‐pentahydroxyisoflavone, 4 = Dihydroquercetin, 5 = 6,8‐Dihydroxykaempferol, 6 = Ellagic acid glucoside, 7 = Nisin, 8 = tetracycline with 32 μg/ml concentration for each. NA: Not active; f: values represent means of three independent replicates ±SD.

Abbreviations: ATCC, American Type Culture Collection; CMCC, China Center of Medicine Culture Collection.

**FIGURE 4:**
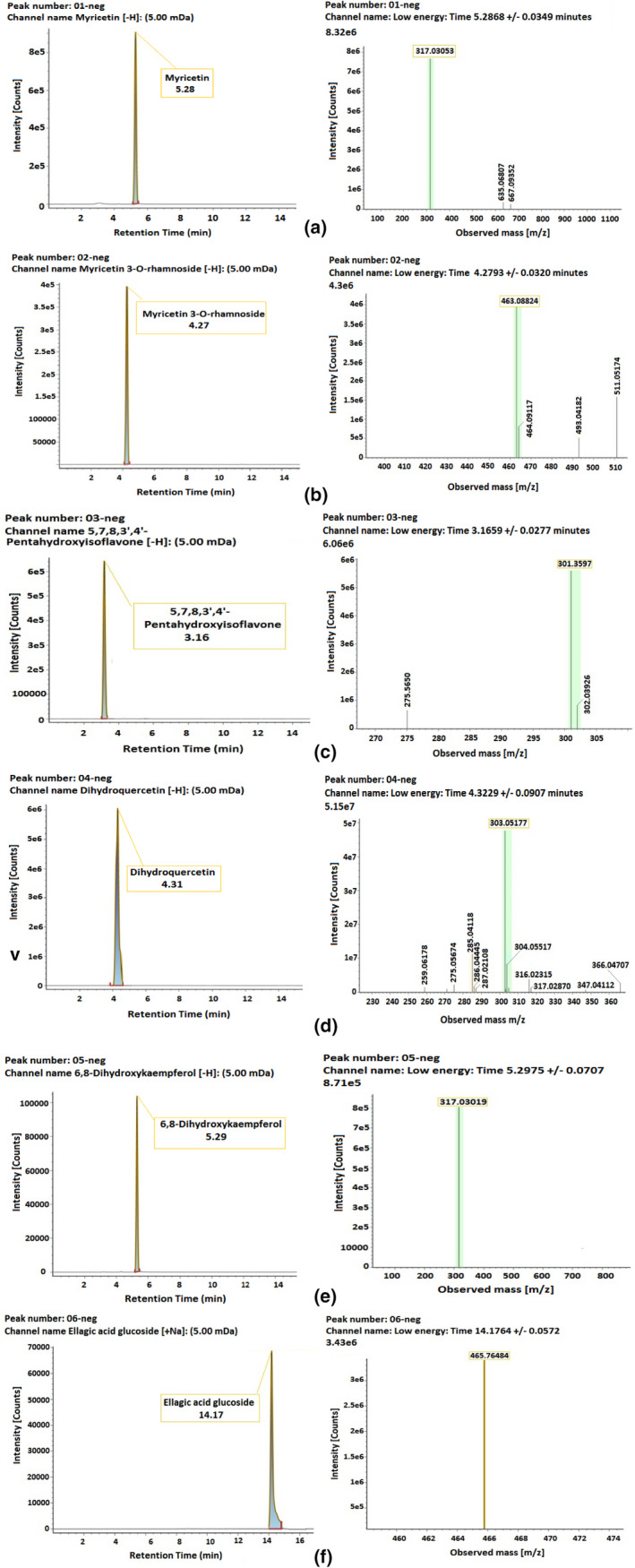
LC‐ESI‐QTOF/MS profiles of six isolated compounds (1–6) from vine tea extract

Furthermore, the LC‐ESI‐QTOF/MS (observed *m*/*z*) spectrum was compared with the calculated *m*/*z* spectrum of compound **3** to detect the error. The LC‐ESI‐QTOF/MS calculated spectra of compound 3 in negative ion mode was 301.03597 m/*z* and observed spectra was 302.04265 m/*z*, which is 301.03597 [M‐H]^‐^ for C_15_H_10_O_7_. Moreover, compound 3 has a maximum ultraviolet (λ_max_) spectrum at 220 nm (Figure 4). This finding is similar to that cited in the tea polyphenol database (TMBD; Xiang et al., 2017; Yue et al., 2014). Therefore, in this study, we only focus on C_15_H_10_O_7_ by testing it in an in vitro assay.

### 3.2 Zone of inhibition

The antimicrobial activity of the six isolated compounds tested against different selected foodborne pathogens is described in Table 2. Different concentrations of six isolated compounds were tested against selected microorganisms and it was found that C_15_H_10_O_7_ (compound 3) possessed the maximum inhibition activity (mm). It was further observed that 32 μg/ml of C_15_H_10_O_7_ was efficient against *S*. *aureus* (CMCCB26003) and *B*. *cereus* (AS11846) with inhibition values of 22.20 ± 0.17 and 25.42 ± 0.12 mm, respectively. The antibacterial activity of C_15_H_10_O_7_ was time‐ and dose‐dependent against gram‐positive and gram‐negative bacteria. The MIC values of C_15_H_10_O_7_ against *S*. *aureus* (CMCCB26003) and *B*. *cereus* (AS11846) were 32 μg/ml (Table 3). Plant‐derived flavonoids are increasingly being studied for their antibacterial properties (Cui, Zeng, et al., 2021). Previously, other flavonoids (dihydromyricetin) in vine tea were isolated and reported for their antimicrobial activity (Liang et al., 2020). It is also reported that presence of a hydroxyl group in an aromatic ring of flavonoids' structure can boost the antimicrobial activity. Whereas, the methylation of active hydroxyl groups may result in the reduction of antimicrobial activity. Furthermore, the lipophilicity of ring A is critical for chalcone action (Senan et al., 2021). Prenyl groups, alkylamino chains, alkyl chains, and nitrogen or oxygen containing heterocyclic moieties are all hydrophobic substituents that increase the activity of flavonoids. Flavonoids are thought to work against bacteria by inhibiting nucleic acid production, cytoplasmic membrane function, energy metabolism, adhesion and biofilm development, suppression of porin on the cell membrane, change in membrane permeability, and reduction in pathogenicity (Xie et al., 2015).

**TABLE 3:** MIC and MBC of 5,7,8,3',4'‐pentahydroxyisoflavone against selected strains of microorganisms

Bacteria	5,7,8,3',4'‐pentahydroxyisoflavone			Control (Tetracycline)		
	ZOI	MIC	MBC	ZOI	MIC	MBC
	(mm)	µg/ml	µg/ml	(mm)	µg/ml	µg/ml
*Bacillus pumilus* CMCC63202	21.37 ± 0.32	64	64	13.47 ± 0.41	64	64
*Bacillus cereus* AS11846	25.42 ± 0.12	32	32	14.12 ± 0.67	64	64
*Escherichia coli* ATCC25922	24.21 ± 0.10	32	32	0	NA	NA
*Staphylococcus aureus* ATCC25923	22.20 ± 0.17	32	32	15.5 ± 0.26	64	64
*Pseudomonas fluorescens* AS11802	22.12 ± 0.25	64	64	NA	NA	NA
*Fusarium graminearum* CICC2021	19.78 ± 0.22	128	256	NA	NA	NA
*Fusarium moniliforme* CICC30174	18.54 ± 0.12	256	512	NA	NA	NA
*Aspergillus flavus* CICC2062	19.78 ± 0.22	256	512	NA	NA	NA

Note

All values represent the mean of three independent experiments ±SD values.

Abbreviations: MBC, minimum bactericidal concentration; MIC, minimum inhibitory concentration; ZOI, zone of inhibition.

**FIGURE 5:**
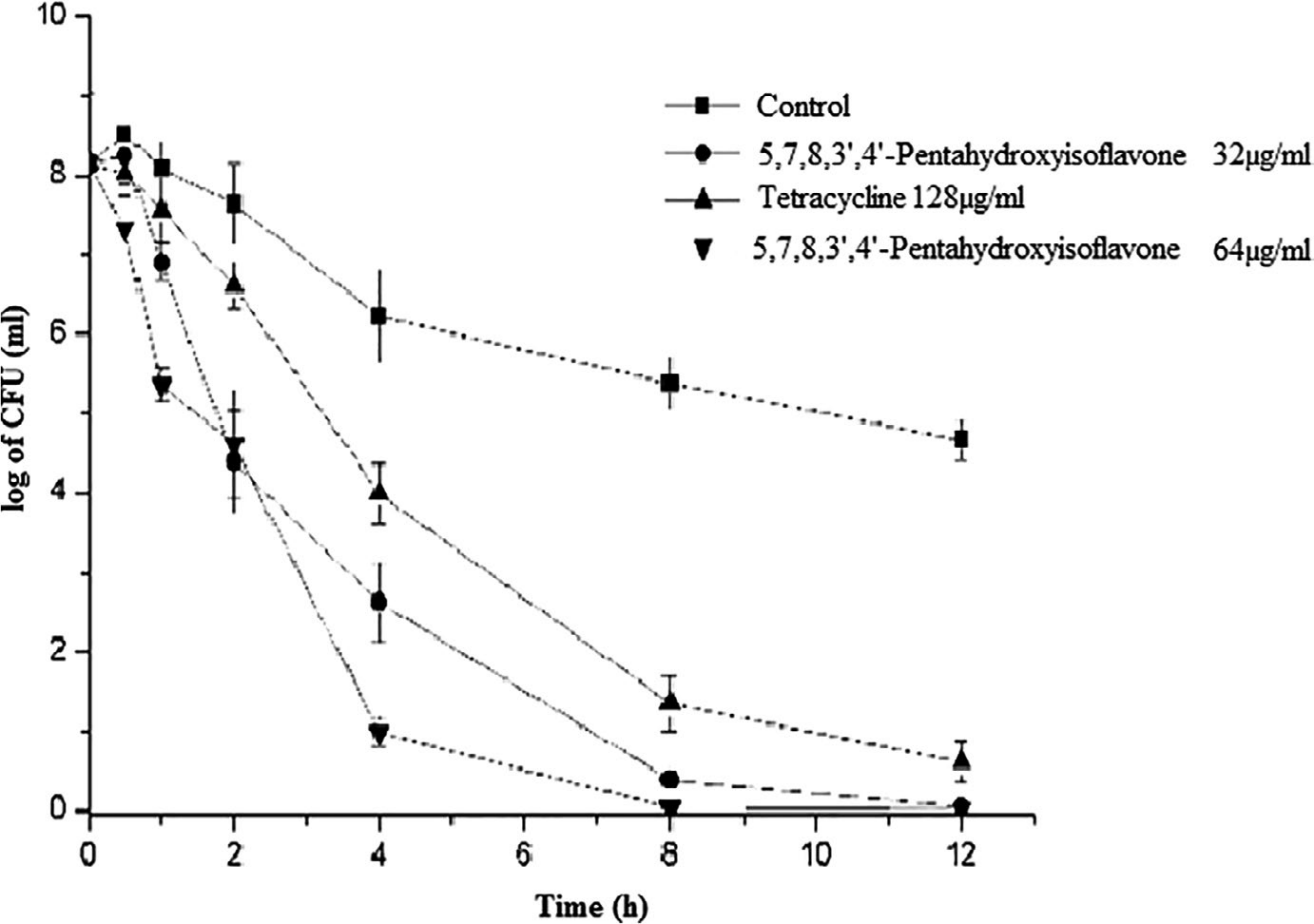
Bactericidal kinetics curve of 5,7,8,3',4'‐pentahydroxyisoflavone (Control, MIC) against *Bacillus cereus* (AS11846)

Previous research has shown that hydroxylation at the fifth and seventh sites is important for flavonol antibacterial activity against bacteria (Echeverría et al., 2017; Umair et al., 2020; Zapadka et al., 2019). Furthermore, another study examined the link between flavonoid structure and antibacterial properties, finding a similar hydroxylation site to be associated with antimicrobial activity (Farhadi et al., 2019) and antibiotic resistance (Abdelgader et al., 2018). During 12 h of exposure to bacterial cells, tetracycline (128 g/ml) can reduce the number of bacterial cells (by 1 log CFU/ml). Simultaneously, the same efficiency may be obtained using C_15_H_10_O_7_ (64 g/ml for 8 h). The viable count was decreased to 1 log CFU/ml, which is half of the tetracycline concentration (Figure 5).

It has been shown that 2R, 3R‐dihydromyricetin (DHM), a flavonoid isolated from vine tea, had good inhibitory action against *S. aureus* (Umair et al., 2020). In terms of the structure–activity connection of flavonoids, very little research has been conducted on the role of the C‐ring in antibacterial activity (Cushnie & Lamb, 2011). Additionally, whether the C_15_H_10_O_7_ structure at the C‐ring is important for antibacterial action remains unknown. On the contrary, studies into the antibacterial mechanism of action of DHM have mostly focused on a common method of action requiring disruption of the cytoplasmic membrane (Cushnie & Lamb, 2011). Furthermore, it has been observed that the antibacterial activity of a particular flavonoid, such as quercetin, is due to a combination of mechanisms (Wu et al., 2018). Previously, a comparable effect of vine tea extract on *S*. *aureus* was observed. Another study reported the antibacterial activity of a flavonol compound isolated from vine tea, in which the activity and mechanism of action of a flavonol compound extracted from *A*. *grossedentata* leaves were studied against foodborne microorganisms. Additionally, the antibacterial activity was tested in relation to pH, thermal processing, and metal ions. The findings indicate that the flavonol molecule has optimal antibacterial activity against five different kinds of foodborne bacteria (*Staphylococcus aureus*, *Bacillus subtilis*, *Escherichia coli*, *Salmonella paratyphi*, and *Pseudomonas aeruginosa*). The antibacterial activity of flavonol compounds was very sensitive to pH, heat processing, and metal ions. The shape of the examined bacteria is altered and the bacteria are more severely harmed when the flavonol compound is exposed for a longer period of time. Additionally, the results of the oxidative respiratory metabolism assay and the cell membrane and wall integrity tests indicated that the death of bacteria caused by flavonol compounds may be due to cell wall lysis, intracellular ingredient leakage, and inhibition of the tricarboxylic acid cycle (TCA) pathway (Xiao et al., 2019). Moreover, the enhanced antimicrobial activity effect of C_15_H_10_O_7_ against gram‐negative molecules might be because of the nature of C_15_H_10_O_7_ in a hydrophilic environment, so it cannot be affected by the lipopolysaccharide molecules of gram‐negative molecules as reported earlier (Xiao et al., 2019). As a result of its hydrophilic nature, it can easily pass through the membrane of its target cell without being blocked, resulting in increased antimicrobial activity. Another study reported better penetration ability of flavonol compounds extracted from vine tea in the hydrophilic environment, thus having no antimicrobial difference when treated with gram‐positive or gram‐negative bacteria (Ameen et al., 2018).

### 3.3 The integrity of the cell membrane

Results indicated that the isolated compound C_15_H_10_O_7_ has the potential to inhibit the bacterial cell. The effect of the isolated compound on the integrity of the cell membrane was noticed when the bacterial suspension was incubated with C_15_H_10_O_7_ at 32 μg/ml for 2 h. A significant increase in absorbance value (OD_260_) was observed in supernatants. This might be due to the leakage of essential cell components which ultimately increases the OD_260_ value (Mohamed et al., 2018). It is widely established that increases in absorbance at OD_260_ reflect nucleic acid and protein leaks from cells and a change in membrane permeability (Liu et al., 2020). Other investigations showed that a chemical with a high affinity for the cell surface and the capacity to change the lipid bilayer was capable of altering the permeability and integrity of the cell membranes and was eventually detrimental to cell membranes (Peng et al., 2020). Taken together, these findings suggest that the isolated chemical examined has the ability to impact cell membranes and limit cell development. A similar dosage and time‐dependent antibacterial action of flavonoids was previously described, with the authors emphasizing the rise in OD_260_ and implying that it was caused by cell membrane leakage (Xie et al., 2015). However, when the supernatant was treated for 4 h, the OD_260_ readings indicated a rapid reaction and a significant increase. Additionally, incubation for 8 h had a significant impact on cell membrane breakdown, as shown by a significant increase in the OD_260_ value (Table 4). However, no significant change in the OD_260_ value was observed between treated and untreated cells. Thus, these findings show that the cell membrane's permeability was enhanced, resulting in the leaking of intracellular contents (nucleic acid and protein) and accounting for the rise in membrane permeability when compound 3 was added. This increase in membrane permeability and alteration of cell integrity are comparable to those seen in prior research (Wu et al., 2017). In addition, smaller charged particles were leaked out before from the cell than the higher charged sized particles such as proteins and nucleic acid (Liao et al., 2014; Zhu et al., 2019). Therefore, it can be inferred that the hydroxylation of C_15_H_10_O_7_ at 5^th^ and 7^th^ positions plays an essential role in antimicrobial activity against bacteria, making it different from the other isolated compounds and giving a solid reason for the current study.

**TABLE 4:** Cell membrane leakage measured at OD_260_ after treating with 5,7,8,3',4'‐pentahydroxyisoflavone (Control, MIC, MBC) against *Bacillus cereus* (AS1.1846)

Cell constituent's release, OD 260 nm	Treatments			
	1 h	2 h	4 h	8 h
Control	0.09 ± 0.02	0.10 ± 0.02	0.10 ± 0.14	0.10 ± 0.54
MIC	0.09 ± 0.11	0.21 ± 0.03	0.24 ± 0.01	0.27 ± 0.03
MBC	0.09 ± 0.03	0.24 ± 0.02	0.29 ± 0.04	0.32 ± 0.01

Note

All values represent means of three replicates ±standard deviation (SD).

### 3.4 Observing morphological changes through SEM

**FIGURE 6:**
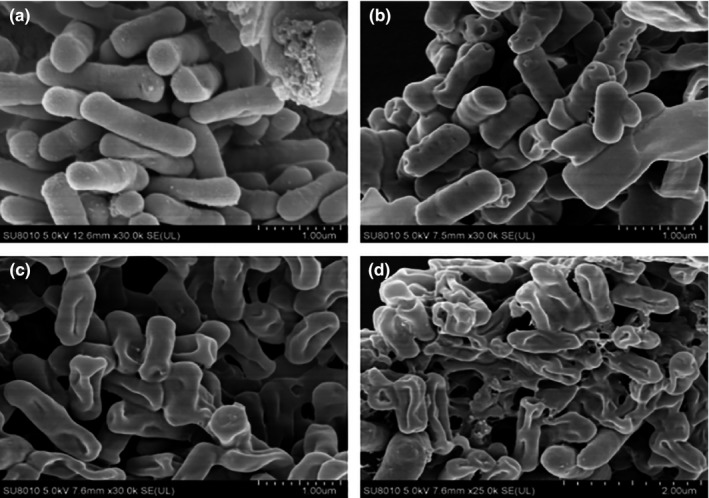
Effects of 5,7,8,3,4‐pentahydroxyisoflavone on *Bacillus cereus* (AS11846) observed with the help of a scanning electron microscope. (a) Results of *B*. *cereus* (AS11846) strains when treated without a sample (Control). (b) Results of *B. cereus* (AS11846) strains when treated with 5,7,8,3,4‐pentahydroxyisoflavone for 1 h at 32 μg/ml. (c) Data for 4 h at 32 μg/ml and (D) for 8 h at 32 μg/ml

**FIGURE 7:**
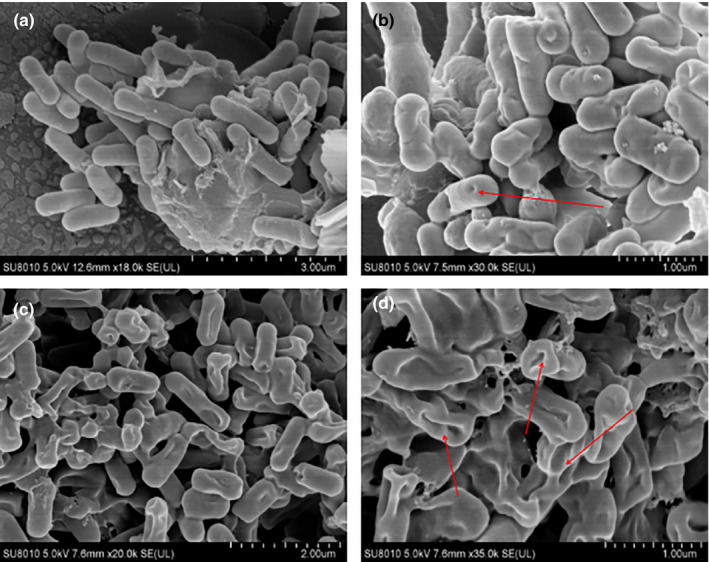
Effects of 5,7,8,3,4‐pentahydroxyisoflavone on *Escherichia coli* (ATCC 25922) observed with the help of a scanning electron microscope. (a) Results of *E*. *coli* (ATCC 25922) when treated without a sample (Control), (b) when treated for 1 h at 32 μg/ml, (c) data for 4 h at 32 μg/ml and (D) for 8 h at 32 μg/ml

The morphological alterations and cell membrane damage in the cell structure were used to confirm the aforementioned results. Scanning electron microscopy has been widely used to investigate structural features of biological materials. The SEM was used to explore the mode of action of C_15_H_10_O_7_ against *B. cereus* (AS11846) and *E. coli* (ATCC25922). The morphological changes in *B. cereus* (AS11846) and *E. coli* (ATCC25922) after treatment with C_15_H_10_O_7_ and without treatment with C_15_H_10_O_7_ (control) are shown in Figures 6(a–d) and 7(a‐d), respectively. As shown in Figures 6a and 7a, untreated cells of *B*. *cereus* (AS11846) and *E*. *coli* (ATCC25922) had a regular form, a red color, striated cells with an intact outer surface and a smooth membrane, and cell structure. This demonstrated that the cells were active and alive despite their minor differences. This little variation in form may be related to a differential in the cell's permeability. According to the study, the permeability of the bacterial cell membrane may have grown over time (Li et al., 2014). When *B*. *cereus* (AS11846) and *E*. *coli* (ATCC25922) bacterial cells were incubated with C_15_H_10_O_7_ at 32 g/ml (1MIC) for 2 h, more irregularities, bleb projections, and concave formation with faster shrinkages were observed on the cell membrane (Figures 6b and 7b).

This may be due to the interaction between ionic proteins and lipids. Although phospholipids are the main constituents of the cell membrane, they perform other functions in addition to creating lipid bilayers (Chi et al., 2020). Acidic phospholipids, for example, create microdomains in the plasma membrane and may interact ionically with proteins through polybasic sequences, potentially affecting the protein's function (Ashraf & Gerke, 2021). Under certain circumstances, these acidic phospholipids may diffuse heterogeneously and form micro‐ or nanodomains (Sarmento et al., 2020). Many proteins have lipid‐binding domains capable of interacting with acidic phospholipids (Sarmento et al., 2020). Additionally, these domains often have well‐defined globular shapes and include positively charged pockets or surfaces that interact with the acidic phospholipids' negatively charged head groups (Kulma & Anderluh, 2021). Positive charges may be provided by basic residues within the domain or by Ca^2+^ ions attached to the domain (Li et al., 2014). Previous research has examined the precise effect of natural antibacterial chemicals (flavonoids) on the morphological characteristics of *E. coli* and *S. aureus* when loaded with nanoparticles (Anwar et al., 2019).

Additionally, the coarse surfaces and even membranes of the cells developed blebs and abnormalities, and a part of the cells turned flat. When *B*. *cereus* (AS11846) and *E*. *coli* (ATCC25922) cells were incubated with C_15_H_10_O_7_ at 32 g/ml (1MIC) for 2 h, transmembrane ion holes formed and the cells began to lose their original shape (rod‐shaped) and became nearly completely flat with very little or no bleb (Figures 6c and 7c). A similar result was previously published, in which the authors documented the development of transmembrane ion holes in the membrane, resulting in the lysis of critical cellular components and nutritional shortage inside the cell (Mironov et al., 2020). During fast shape transition, the quantity and size of lipid heads within lipid leaflets (i.e., owing to a change in lipid head charge) modify the geometrical characteristics of organelles, most notably their membrane curvature. Protein insertion into a lipid bilayer and the form of protein transmembrane domains also influence the transmembrane asymmetry between the surface areas of the membrane's luminal and cytosol leaflets. Where a particular shape of lipid molecule is not prevalent, the form of lipids (cylindrical, conical, or wedge‐like) has a lesser role in regulating membrane curvature, owing to the flexibility of their acyl chains and their great capacity to diffuse (Mironov et al., 2020). Therefore, any changes in the protein transmembrane domains also affect the transmembrane asymmetry and eventually cause cell surface deformation (Ahmed et al., 2021). The cell structure was completely flat with no bleb and completely lost its formal shape when *B. cereus* (AS11846) and *E. coli* (ATCC25922) cells were incubated with C_15_H_10_O_7_ at 32 μg/ml (1 × MIC) for 8 h (Figures 6d and 7d).

The present research hypothesized that the bactericidal impact of C_15_H_10_O_7_ on gram‐positive and gram‐negative bacteria was due to changes in cell permeability, which may alter the interaction of membrane proteins and lipids, disrupting the ionic protein–lipid link (Li et al., 2014). Thus, it has an effect on, and disrupts the structure and function of, proteins (Ashraf & Gerke, 2021). These findings corroborate the observation that phospholipids are a major component of the cell membrane (Chi et al., 2020). They provide a functional purpose for certain acidic phospholipids by forming a microdomain in the plasma membrane and interacting with protein ionically through polybasic sequences as reported by Sarmento, Hof, et al. (2020) and Sarmento, Ricardo, et al. (2020). Additionally, the existence of transmembrane holes indicated the formation of ion channels, which resulted in cell membrane rupture, as shown in Figures 5c and 6c for *B*. *cereus* (AS11846) and *S*. *cereus* (ATCC25922), respectively. Another study reached a similar conclusion, where the electrostatic interaction between the positively charged guanidinium head group and the negatively charged phospholipid head group in the cytoplasmic membrane resulted in the formation of a hydrophobic membrane core (Ma et al., 2017).

Nisin targets the membrane‐bound cell wall, interacts with the precursor of lipid II, and retards the outer cell membrane (peptidoglycan) (Panina et al., 2020). This results in the formation of pores on the cell wall, which eventually cause cell lysis (Gharsallaoui et al., 2016). The hydrophobic residues of C_15_H_10_O_7_ may align and bind to the hydrophobic membrane core to form a hydrophilic channel, causing functional changes in proteins, nucleic acids, and potassium ions. Lantibiotics (e.g., nisin) are supposed to be natural substances that efficiently suppress bacterial populations, but their therapeutic use is very restricted. Nisin binds to the membrane‐embedded precursor of the cell wall, lipid II, by trapping its pyrophosphate group, which is unlikely to evolve and therefore offers an attractive pharmacological target (Panina et al., 2020). A similar phenomenon has been reported in a previous study, where the antibacterial activity and mechanism of monolauroyl‐galactosylglycerol against *B. cereus* was explored (Diao et al., 2018).

Based on the above findings, we hypothesize that C_15_H_10_O_7_ alters the permeability of gram‐positive and gram‐negative bacteria by affecting membrane protein and lipid interactions, thereby disrupting the ionic protein–lipid bond and ultimately disrupting protein structure and function. However, the presence of a higher hydrophobic membrane core in gram‐positive bacteria also showed an altered pathway for cell inhibition by the binding of lipid II. Thus, the current study has depicted that C_15_H_10_O_7_ has solid antibacterial activity as observed in vitro. Further research is needed to develop natural antibacterial drugs as a better alternative to antibiotics against gram‐positive and gram‐negative bacteria.

## 4 CONCLUSION

This study explored the antimicrobial properties of vine tea (*A*. *grossedentata*) against disease‐causing foodborne pathogens. Successful RP‐HPLC isolation of six bioactive molecules was carried out, which was further confirmed through LC‐MS/MS. Among these bioactive compounds, C_15_H_10_O_7_ was found to have intense antimicrobial activity against *B*. *cereus* and *S*. *aureus*. A dose‐dependent effect on the bactericidal kinetics with a higher degree of OD_260_ absorbance altered the membrane permeability. The alteration in membrane permeability could facilitate the transport of cell material. SEM analysis further confirmed the formation of ion channels and transmembrane pores that result in increased membrane permeability and cytoplasm leakage. However, the presence of a higher hydrophobic membrane core in gram‐positive bacteria also suggested that another action mode of C_15_H_10_O_7_ is involved in the bio‐static pathway and affect the ionic protein–lipid bond, which ultimately disturbs the protein structure and its normal functioning. The potent antimicrobial properties of vine tea (C_15_H_10_O_7_) need to be explored further for the development of effective drugs to counter bacterial pathogens.

## CONFLICT OF INTEREST

The authors declare that they have no competing interests.

## ETHICS APPROVAL AND CONSENT TO PARTICIPATE

Not applicable.

## CONSENT FOR PUBLICATION

Not applicable.

## Data Availability

The datasets used and/or analyzed during this study are available from the corresponding author upon reasonable request. However, all the data explained here are enough to support the results and findings of the manuscript.
